# Supplementary material for: No-reference quality assessment for image-based assessment of economically important tropical woods
Source: PLoS One. 2020 May 19;15(5):e0233320. doi: 10.1371/journal.pone.0233320 (PMC7236984; doi:10.1371/journal.pone.0233320)
Supplement: Attachment — Submitted filename: Response to Reviewers.docx [file pone.0233320.s001.docx]

5 March 2020

Dear Editor and Reviewers,

Thank you very much for considering our manuscript entitled “**No-reference quality assessment for image-based assessment of economically important tropical woods**”. We really appreciate your valuable suggestions and comments which help us to enhance the quality of our paper. We have revised the manuscript accordingly, with details below:

*Reviewers' comments:*

1. *When submitting your revision, we need you to address these additional requirements.*

*Please ensure that your manuscript meets PLOS ONE's style requirements, including those for file naming. The PLOS ONE style templates can be found at http://www.journals.plos.org/plosone/s/file?id=wjVg/PLOSOne_formatting_sample_main_body.pdf and*

[*http://www.journals.plos.org/plosone/s/file?id=ba62/PLOSOne_formatting_sample_title_authors_affiliations.pdf*](http://www.journals.plos.org/plosone/s/file?id=ba62/PLOSOne_formatting_sample_title_authors_affiliations.pdf)

- The manuscript has been revised according to the PLOS ONE's style.

2. *Please amend your Data availability statement to provide links/URLs to how other researchers may access the images and data used in this study.*

- The images and data used in this study are publicly available in [https://github.com/Heshalini/Wood-Image-Quality/tree/b03346da9d1efb887ec6d390f235822cc9470f49.](https://github.com/Heshalini/Wood-Image-Quality/tree/b03346da9d1efb887ec6d390f235822cc9470f49.%20)

3. *We suggest you thoroughly copyedit your manuscript for language usage, spelling, and grammar. If you do not know anyone who can help you do this, you may wish to consider employing a professional scientific editing service.*

- The manuscript has been proofread and edited by professional proofreading and editing service, Proofreading by A UK PhD.

4. *We note that Figures 1-8 in your submission contain copyrighted images. All PLOS content is published under the Creative Commons Attribution License (CC BY 4.0), which means that the manuscript, images, and Supporting Information files will be freely available online, and any third party is permitted to access, download, copy, distribute, and use these materials in any way, even commercially, with proper attribution.*

- Figs 1-8 were generated using the publicly available wood images database owned by Mr Eric Meier. Permission to publish the figures has been obtained from Mr Eric Meier and the completed Content Permission Form and e-mail from Mr Eric which mentioned that he permits the usage of the wood images from his website are uploaded as an "Other" file with this submission.

5. *Reviewer 1:*

*In this paper, the authors proposed a no-reference IQA (NR-IQA) metric to assess the quality of wood images, Wood NR-IQA (WNR-IQA). Support Vector Machine (SVM) Regression (SVR) was trained using Generalized Gaussian Distribution (GGD) and Asymmetric Generalized Gaussian Distribution (AGGD) features calculated for wood images and the mean opinion score (MOS) obtained from the subjective evaluation. Compared to other six metrics, namely Blind/Referenceless Image Spatial Quality Evaluator (BRISQUE) and five Full Reference-IQA (FR-IQA) metrics known as MSSIM, SSIM, FSIM, IWSSIM and GMSD, the proposed WNR-IQA had better performance in PLCC and RMSE values. Furthermore, it is meaningful that the proposed metric does not require a “perfect” reference image in order to evaluate the images.*

*But, this manuscript in this current form has some problems as follows:*

i. *The introduction is not enough because the references for the current researches are too few while the related analyses are not profound.*
- Explanation on current researches done on CNN based NR-IQAs, deepIQA [12] and Deep Bilinear Convolutional Neural Networks (DB-CNN) [14] were added in **line 81-106 of the Introduction section***.* DeepIQA and DB-CNN are CNN based NR-IQAs where deepIQA is trained end-to-end and involves 10 convolutional layers, 5 pooling layers for feature extraction and 2 fully connected layers for regression [12] while DB-CNN is trained by two sets of features namely, CNN for synthetic distortions (S-CNN) and VGG-16, that are bi-linearly pooled to predict the quality of the image [14]. However, CNN based NR-IQA model requires a very large training database as a limited number of labelled training data often leads to overfitting problem in CNN [14].
ii. *The metric most relevant to the proposed metric is the BRISQUE. But the authors did not show the differences between them, especially the difference in image features adoption and the difference in the procedure of these two metrics. Please add relevant instructions to highlight the innovation of this paper.*
- The innovation of the proposed WNR-IQA model is that it is a No Reference – Image Quality Assessment (NR-IQA) model designed specifically for wood images. WNR-IQA is motivated by NR-IQA model which is independent of reference natural images. In fact, for practicality, good reference images especially given the nature of the wood industry (dusty environment and poor illumination) are not easily available. These were written in **line 78-80 of the Introduction section**.
- The proposed model uses a similar concept designed for natural images, with mathematical models that consider several factors like contrast, luminance, image feature, and Natural Scene Statistics (NSS). The key difference between the BRISQUE and WNR-IQA is that the BRISQUE was developed by training the SVR using the image features computed for natural images; whereas the WNR-IQA used the wood images for this calculation. These were written in **line 277-282 of the Regression module section**. Their differences were also illustrated in **Fig 6, Regression module section**.
iii. *In Eq. (4) and (7), what does the “x” mean?*
- Eq. (4) is the equation to calculate GGD features. *x* in Eq. (4) is the Mean Subtracted Contrast Normalized (MSCN), $\hat{I}\left( m,n \right)$ of wood images. **This was written in Eq. (5), The features of GGD and AGGD section.**
- Eq. (7) (in previous manuscript version) is now Eq. (16) which is the equation to calculate AGGD features. *x* in Eq. (16) is the pairwise products of MSCN coefficients which are computed along four orientation, $H_{1},V_{1}, D_{1}and D_{2}$. **This was written in Eq. (17), The features of GGD and AGGD section**
  iv. *In section “Results and Discussions”, the authors have only considered the Gaussian white noise and motion blur. Why the mixture of these two noises were ignored in simulating the image distortions encountered in the industrial setting?*
- All the ten wood images in the second dataset have been distorted with the mixture of Gaussian white noise and motion blur. The images which were distorted by Gaussian white noise were further distorted with motion blur, i.e. images distorted with $\sigma_{GN}$ = 10 were further distorted with $\sigma_{MB}$ = 2, 4, 6, 8 and 10 and the same procedure were repeated for images distorted with $\sigma_{GN}$ = 20, 30, 40 and 50. In total, this dataset comprises of 360 wood images. These were written in **line 314 – 317 of the Performance evaluation section**. The results obtained for the mixture of Gaussian white noise and motion blur can be found in **line 327– 365 of the Results and discussions section**.
  v. *In section “Results and Discussions”, why the ten wood images mentioned in “wood images”, namely Avodire, Bocote, Butternut, Basswood, Dogwood, Laurel Blanco, Katalox, and Keruing are not taken into experiments? They are replaced by other ten species, why?*
- The ten wood images mentioned in the wood images section are used to train and test the SVR model. These images were distorted with Gaussian White Noise and Motion Blur and were used to train and test the SVR model for 100 times where 100 PLCC and RMSE values were obtained and the median of these values were chosen in order to select the optimized cost parameter, C, and width parameter, g, of the SVR model. These are written in **line 269-293 of the Regression module section**. The median values of PLCC and RMSE were also mentioned in these lines.
- The performance of the proposed WNR-IQA metric is evaluated using the second dataset which also comprises of different ten wood images. Therefore, the results obtained for these images were included and discussed in the Results and Discussion section. The second dataset is used to evaluate the performance of WNR-IQA metric instead of the ten wood images mentioned in the wood images section (used to train SVR) to avoid any bias. These are written in **line 295-299 of the Performance evaluation section**.
  vi. *In section “Relationship between MOS and Proposed WNR-IQA, BRISQUE, FR-IQAs”, there is no detailed explanation about Fig 8. In addition, pictures shown in Fig 8a-u are blurred. It is unacceptable.*
- Fig 8a-u which shows the distribution of the WNR-IQA, BRISQUE, FR-IQAs has been removed due to the poor quality of the images. Moreover, the relationship between MOS and Proposed WNR-IQA, BRISQUE, FR-IQAs is shown through the PLCC and RMSE values in **Table 2, Relationship between MOS and proposed WNR-IQA, BRISQUE, deepIQA, DB-CNN, FR-IQAs section.**
  vii. *Please clearly explain the reasons in section “Results and Discussions” based on the experimental results, why the proposed metric WNR-IQA is superior to the other six metrics? And why other related metrics have lower performance?*
- The WNR-IQA was compared with BRISQUE, deepIQA, DB-CNN, five FR-IQAs: SSIM, MS-SSIM, FSIM, IW-SSIM and GMSD obtained for the second dataset. WNR-IQA outperforms BRISQUE, deepIQA, DB-CNN and FR-IQAs as it is tailored for wood images where the model has been trained with GGD and AGGD features and MOS obtained for wood images specifically unlike BRISQUE, deepIQA, DB-CNN and FR-IQAs which were designed by considering the features and similarities of features, luminance, contrast and structure of natural images. Furthermore, WNR-IQA is better than the FR-IQAs as it does not require a perfect reference image. These were written **in line 347 – 365 of the Relationship between MOS and proposed WNR-IQA, BRISQUE, deepIQA, DB-CNN, FR-IQAs section**.
  viii. *The 18 parameters of GGD and AGGD for wood images are keys for proposed WNR-IQA. But there are no equations or detailed instructions to show how to calculate these parameters in this paper. That should not be neglected or omitted.*
- The equations and detailed instruction for GGD and AGGD parameters were added and can be found in **line 182-234 and Table 1 of The features of GGD and AGGD section**.
  ix. *English expressions need to be carefully checked out. For example, in the first paragraph of Introduction, there exist the following sentence (noting “it high strength and density”):*

*“Rosewood (Dalbergia sp.) is another expensive wood, sought after for instrument making and flooring due to its high strength and density.”*

*Besides, at line 111 to 112, there is a sentence (noting “were the distorted”):*

*“These ten reference wood images were distorted by Gaussian white noise and motion blur, which represent image distortions typically encountered in the industrial setting.”*

- The above sentences were corrected and the manuscript has been proofread and edited by professional proofreading and editing service, Proofreading by A UK PhD.

6. *Reviewer #2: The authors presented a no-reference IQA metric to assess the quality of wood images. Some experiments are done and compared with some other metrics. The results are rather OK. However, I can not find the innovative points in the paper since the authors just utilize the regression module to assess the quality of wood images. Furthermore, many state-of-the-art references are not cited. The experiments just compared with traditional methods, and many recent methods, especial some deep learning-based methods such as Hallucinated-IQA and deepIQA, are not used to compare.*
   - The innovation of the proposed WNR-IQA model is that it is a No Reference – Image Quality Assessment (NR-IQA) model designed specifically for wood images. WNR-IQA is motivated by NR-IQA model which is independent of reference natural images. In fact, for practicality, good reference images especially given the nature of the wood industry (dusty environment and poor illumination) are not easily available. These were written in **line 78 - 80 of the Introduction section**.
   - The proposed model uses a similar concept designed for natural images, with mathematical models that consider several factors like contrast, luminance, image feature, and Natural Scene Statistics (NSS). The key difference between the BRISQUE and WNR-IQA is that the BRISQUE was developed by training the SVR using the image features computed for natural images; whereas the WNR-IQA used the wood images for this calculation. These were written in the **line 277 - 282 of the Regression module section**. Their differences were also illustrated in **Fig 6**.
   - The WNR-IQA was also compared with deepIQA and DB-CNN obtained for the second dataset. Results shows that WNR-IQA outperforms these two NR-IQAs as well. These were written in **line 347 – 365 of the** **Relationship between MOS and proposed WNR-IQA, BRISQUE, deepIQA, DB-CNN, FR-IQAs section**.

*Minor points:*

*What’s mean of \in -SVR in Line 239?*

- $\in-$SVR is epsilon-SVR which is a type of SVR model. This was written in **line 269 of the Regression module section**.

*In equ. 19, it’s better to add subscript k in MOS since you calculate the MOS of the k^{th} image.*

- Subscript *k* was replaced with *p* to avoid confusion with the window sizes in Eq. (2) and (3). Subscript *p* was added in MOS equation in **Eq. (21), MOS section**.

Thank you for your kind consideration of our work.

Yours Faithfully,

**NORRIMA MOKHTAR**

Senior Lecturer

Department of Electrical Engineering

Faculty of Engineering

University of Malaya

50603 Kuala Lumpur

Malaysia
