# Supplementary material for: No-reference quality assessment for image-based assessment of economically important tropical woods
Source: PLoS One. 2020 May 19;15(5):e0233320. doi: 10.1371/journal.pone.0233320 (PMC7236984; doi:10.1371/journal.pone.0233320)
Supplement: Attachment — Submitted filename: Response to Reviewers.docx [file pone.0233320.s002.docx]

25 April 2020

Dear Editor and Reviewers,

Thank you very much for considering our manuscript entitled “**No-reference quality assessment for image-based assessment of economically important tropical woods**”. We really appreciate your valuable suggestions and comments which help us to enhance the quality of our paper. We have revised the manuscript accordingly, with details below:

*Reviewers' comments:*

1. *Reviewer #2: My comments are responded by authors carefully. The manuscript is improvement significantly. However, the model description is still not clear enough. Please give some explanation the difference between "natural image" and "wood image" . In Line 216, a parameter \eta is missed*
   - The features of GGD and AGGD section was revised to make the model description clear. The content of the features of GGD and AGGD section was arranged to be in a flow to make the model description clear. Furthermore, line 227- 228 was revised by adding the following statement:

“The AGGD parameters, $\eta, v,\sigma_{l}^{2},\sigma_{r}^{2}$ were calculated throughout $H_{1}$*,* $V_{1}$*,* $D_{1}$ and $D_{2}$ orientations as shown in equation (17) and this forms 16 parameters of AGGD.”

Table 1 which explains the 18 GGD and AGGD parameters was corrected as there was an error previously. The revised content can be found in **line 146 – 250.**

Fig 6 which shows the differences between BRISQUE and WNR-IQA was revised where the MSCN for the images was added into the flowchart to make the flowchart of the model clear.

- The natural and wood images were explained in **line 283-285, Regression module section**. Natural images are any natural light images which are captured by an optical camera without any pre-processing. While, wood images are captured using a portable camera which has ten times magnification lens.
- The missing parameter *\eta* in line 216 has been revised. The revised line can be found in **line 224, The features of GGD and AGGD section**.

The summary of the revision made after the reviewer’s comment is shown in the following table:

| **Reviewer’s Comment** | **Before Revision** | **After Revision** |
| --- | --- | --- |
| *However, the model description is still not clear enough* |  | Figure caption for Fig 4 was rearranged in the text of the manuscript, immediately following the paragraph in which the figure is first cited. |
|  |  | Figure caption for Fig 5 was rearranged in the text of the manuscript, immediately following the paragraph in which the figure is first cited. |
|  |  |  |
|  |  |  |
|  |  |   Fig 6 which shows the differences between BRISQUE and WNR-IQA was revised where the MSCN for the images was added into the flowchart to make the flowchart of the model clear. |
| *Please give some explanation the difference between "natural image" and "wood image"* |  |  |
| *In Line 216, a parameter \eta is missed* |  |  |

Thank you for your kind consideration of our work.

Yours Faithfully,

**NORRIMA MOKHTAR**

Senior Lecturer

Department of Electrical Engineering

Faculty of Engineering

University of Malaya

50603 Kuala Lumpur

Malaysia
